# Repeated Intake of Grapefruit Juice Inhibits CYP2B6, CYP2C9, CYP2C19, and CYP3A4 while Lingonberry Powder Does Not Induce Major CYP Enzymes in Humans

**DOI:** 10.1002/cpt.70165

**Published:** 2025-12-14

**Authors:** Laura Aurinsalo, Outi Lapatto‐Reiniluoto, Mika Kurkela, Mikko Neuvonen, Eeva Moilanen, Mikko Niemi, Aleksi Tornio, Janne T. Backman

**Affiliations:** ^1^ Department of Clinical Pharmacology University of Helsinki Helsinki Finland; ^2^ Individualized Drug Therapy Research Program, Faculty of Medicine University of Helsinki Helsinki Finland; ^3^ Department of Clinical Pharmacology, HUS Diagnostic Center Helsinki University Hospital Helsinki Finland; ^4^ HUS Pharmacy Helsinki University Hospital Helsinki Finland; ^5^ The Immunopharmacology Research Group, Faculty of Medicine and Health Technology Tampere University, and Tampere University Hospital Tampere Finland; ^6^ Integrative Physiology and Pharmacology, Institute of Biomedicine University of Turku Turku Finland; ^7^ Unit of Clinical Pharmacology Turku University Hospital Turku Finland

## Abstract

Grapefruit juice is a well‐established inhibitor of cytochrome P450 (CYP) 3A4, but its effects on other CYP enzymes or organic anion transporting polypeptides (OATPs) are not fully characterized in humans. Recently, lingonberry powder was shown to induce murine CYP enzymes. We investigated the effects of lingonberry powder and grapefruit juice on seven CYP enzymes and two OATPs. Eleven healthy volunteers received three pretreatments three times per day: water for 1 day (control), lingonberry powder for 9 days, and grapefruit juice for 3 days. CYP index drugs (caffeine/CYP1A2, bupropion/CYP2B6, repaglinide/CYP2C8, flurbiprofen/CYP2C9, omeprazole/CYP2C19, dextromethorphan/CYP2D6, midazolam/CYP3A4, and simvastatin/CYP3A4) were administered orally on the study day of each pretreatment (day 1, 10, and 3, respectively). Venous blood samples were collected until 23 hours postdose. The concentrations of index drugs, their metabolites and endogenous OATP1B1 and OATP1B3 biomarkers glycochenodeoxycholate 3‐O‐glucuronide (GCDCA‐3G) and glycochenodeoxycholate 3‐sulfate (GCDCA‐3S), respectively, were quantified. Grapefruit juice expectedly increased the AUC_0–23h_ values of the CYP3A4 index drugs midazolam and simvastatin (*P* < 0.01). Additionally, grapefruit juice decreased the hydroxybupropion/bupropion (CYP2B6), 4′‐hydroxyflurbiprofen/flurbiprofen (CYP2C9), 5′‐hydroxyomeprazole/omeprazole (CYP2C19), and 1′‐hydroxymidazolam/midazolam AUC_0–23h_ ratios to 0.57‐fold (90% confidence interval: 0.45–0.74), 0.78‐fold (0.69–0.87), 0.43‐fold (0.36–0.52), and 0.72‐fold (0.63–0.84) of control, respectively (*P* < 0.01). Lingonberry pretreatment did not change any CYP indices. GCDCA‐3G and GCDCA‐3S concentrations were unaffected by grapefruit juice or lingonberry pretreatment. Collectively, our findings indicate that in addition to inhibiting CYP3A4, repeated grapefruit juice intake causes clinically relevant inhibition of CYP2B6, CYP2C9, and CYP2C19, revealing previously underappreciated interaction risks. Conversely, lingonberry powder is unlikely to induce CYP enzymes in humans.

**Study Highlights**

**WHAT IS THE CURRENT KNOWLEDGE ON THE TOPIC?**

Grapefruit juice is a well‐established inhibitor of cytochrome P450 (CYP) 3A4 in the intestinal wall. This effect insufficiently explains the interactions of grapefruit juice with some drugs that are not sensitive to CYP3A4 inhibition. Another food product, lingonberry powder has induced CYP enzymes in mice but the effects on human CYP enzymes are unknown.

**WHAT QUESTION DID THIS STUDY ADDRESS?**

We investigated the inhibitory effects of grapefruit juice and the possible inducing effect of lingonberry powder on seven of the most important human CYP enzymes and two organic anion transporting polypeptides (OATPs) by using a previously developed phenotyping cocktail with eight index drugs for CYP enzymes and endogenous biomarkers for OATP1B1 and OATP1B3.

**WHAT DOES THIS STUDY ADD TO OUR KNOWLEDGE?**

In addition to inhibiting CYP3A4, repeated grapefruit juice intake inhibits CYP2B6, CYP2C9, and CYP2C19. On the contrary, typical intake of lingonberry powder does not induce CYP enzymes. Moreover, grapefruit juice and lingonberry powder have no effect on OATP1B1 or OATP1B3 activities.

**HOW MIGHT THIS CHANGE CLINICAL PHARMACOLOGY OR TRANSLATIONAL SCIENCE?**

Contrary to what has been previously thought, grapefruit juice inhibits CYP2B6, CYP2C9, and CYP2C19 in addition to CYP3A4. Thus, caution is warranted with repeated grapefruit juice consumption during the intake of drugs that are metabolized by these CYP enzymes. This knowledge should also be used when evaluating the effects of grapefruit juice in clinical drug interaction trials.

Drug–drug interactions are a well‐known challenge during pharmacotherapy, but clinically relevant interactions can also occur between food substances and drugs. Grapefruit juice is probably the most well‐known perpetrator in food–drug interactions as grapefruit juice inhibits the cytochrome P450 (CYP) 3A4‐mediated metabolism of drugs in the intestinal wall and can thereby increase drug exposure several fold.^1^, ^2^ Grapefruits contain furanocoumarins, for example, bergamottin and dihydroxybergamottin, which are thought to explain the CYP3A4 inhibitory effect. In addition, grapefruit juice, among some other fruit juices, has been shown to reduce the absorption of several drugs, possibly via inhibition of intestinal influx transporters.^3^

Some of the reported effects of grapefruit juice are poorly explained by inhibition of CYP3A4 in the small intestine. For example, grapefruit juice pretreatment decreased dramatically, by over 80%, the concentrations of the active metabolite of clopidogrel, resulting in a decreased platelet‐inhibitory effect.^4^ The role of CYP3A4 is relatively small in the formation of this metabolite, with other enzymes, particularly CYP2C19, playing a major role.^5^ Additionally, the concentrations of sertraline, which is metabolized by CYP2B6, CYP2C19, and CYP3A4, were significantly increased after grapefruit juice consumption.^6^, ^7^ Considering these previous findings, the clinical effects of grapefruit juice on CYP enzymes beyond CYP3A4 warrant more investigation.

Recently, lingonberry powder supplementation was found to inhibit high‐fat diet‐enhanced expression of multiple inflammatory factors in the liver in a murine model of obesity.^8^, ^9^ In addition, the mean expression of *Cyp3a11* mRNA was increased 2.85‐fold after a lingonberry powder‐containing diet, while those of *Cyp2c55*, *Cyp2c29*, and *Cyp3a59* were increased 2.22‐fold, 1.75‐fold, and 1.55‐fold, respectively, and that of *Cyp46a1* was decreased by 65%.^9^ The murine Cyp3a11 enzyme shares some properties with human CYP3A4, raising a question of whether lingonberry supplementation could induce human CYP3A4 activity, the most important drug‐metabolizing enzyme in humans.^9^ Moreover, solute carrier organic anion transporter family member 1a4 (*Slco1a4*) expression was increased 1.58‐fold after lingonberry powder supplementation, showing possible induction of this organic anion transporter as well.^9^ The exact lingonberry compounds inducing expression of murine *Cyp* and *Slco* genes are not known. Lingonberries are commonly used in the Scandinavian diet as fresh or frozen in many traditional foods and also as air‐dried powders, and according to the recent experimental studies, may inhibit obesity‐associated inflammatory and metabolic adverse effects.^8^, ^9^ Thus, the effect of lingonberry on human CYP enzymes and organic anion transporting polypeptides (OATPs) warrants further investigation.

In this study, our aim was to determine the inhibitory effects of grapefruit juice and possible inducing effects of lingonberry powder on seven major CYP enzymes and OATP1B1 and OATP1B3 with a previously developed phenotyping cocktail and sensitive biomarkers.^10^ As we expected to observe the most significant effect on intestinal CYP3A4, we included an additional sensitive index drug for intestinal CYP3A4 activity, simvastatin, in the cocktail.^11^, ^12^, ^13^

## METHODS

### Study design

**Figure 1:**
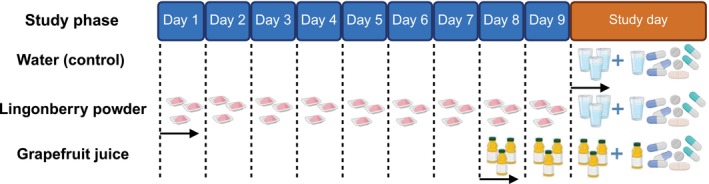
In a controlled clinical trial with three phases, 11 healthy subjects received the following pretreatments in fixed order: 200 mL water at 8 a.m., 12 a.m., and 8 p.m. on the study day (phase I), 5 g lingonberry powder three times per day for 9 days followed by 200 mL water at 8 a.m., 12 a.m., and 8 p.m. on the study day (phase II), and 200 mL grapefruit juice at 8 a.m., 12 a.m., and 8 p.m. for 3 days, the third of which was the study day (phase III). Index drugs (50 mg caffeine/CYP1A2, 20 mg bupropion/CYP2B6, 0.05 mg repaglinide/CYP2C8, 10 mg flurbiprofen/CYP2C9, 10 mg omeprazole/CYP2C19, 10 mg dextromethorphan/CYP2D6, 1 mg midazolam/CYP3A4, and 10 mg simvastatin/CYP3A4) were administered orally at 9.00 a.m. on the study day with 250 mL of water in phases I and II and with 250 mL of grapefruit juice in phase III. Created in BioRender. Aurinsalo, L. (2026) https://BioRender.com/zvv7lid.

This open‐label clinical trial included three study phases that each participant underwent in a fixed order (**Figure** **1**). There was a washout period of at least 3 weeks between different study days. The pretreatment included water, lingonberry powder (Puolukkajauhe, Kiantama Oy, Suomussalmi, Finland), or grapefruit juice (Tropicana Golden Grapefruit, PepsiCo BeLux B.V., Zaventem, Belgium) depending on the study phase (**Figure** **1**). As for the index drugs, the Helsinki and Uusimaa Hospital District Pharmacy manufactured 0.05 mg repaglinide capsules, 20 mg bupropion capsules, and 10 mg flurbiprofen capsules from the following commercially available preparations: Repaglinide Krka 0.5 mg tablet (KRKA, Novo mesto, Slovenia), Bupropion Hydrochloride Tablet 100 mg (Heritage Pharmaceuticals Inc. Eatontown, NJ), and Cebutid 50 mg tablet (Almirall SA, Barcelona, Spain), respectively, with the addition of microcrystalline cellulose for repaglinide and bupropion capsules and lactose for flurbiprofen capsules. The other index drugs were administered as commercially available preparations: 50 mg caffeine (exact half of Caffeine 100 mg tablet, University Pharmacy, Helsinki, Finland), 10 mg omeprazole (gastro‐resistant Losec Mups 10 mg tablet, Astra Zeneca, Cambridge, United Kingdom), 10 mg dextromethorphan (5.0 mL of Rometor Ratiopharm 2 mg/mL oral solution, Ratiopharm, Ulm, Germany), 1 mg midazolam (0.50 mL of Midazolam‐ratiopharm 2 mg/mL oral solution, Ratiopharm, Ulm, Germany), and 10 mg simvastatin (Simvastatin Krka 10 mg tablet, KRKA, Novo mesto, Slovenia).

On study days, venous blood samples from all participants were drawn from forearm vein cannulas before the administration of water or grapefruit juice at 8 a.m. and 0.5, 1, 1.5, 2, 3, 4, 6, 8, 12, and 23 hours after the administration of the index drugs at 9 a.m. for determination of index drug and metabolite concentrations. Standardized breakfast was served 1 hour, lunch 3 hours, and snacks 7 and 9 hours after the administration of index drugs. For safety, blood glucose levels were monitored with a CareSens Dual point‐of‐care blood glucose meter (i‐SENS Inc. Seoul, South Korea) at 2‐hour and 4‐hour venous blood sampling timepoints after the administration of index drugs.

The use of lingonberry, any food products rich in berries, grapefruit juice, pomelo, and other related fruits was prohibited from 1 week before and throughout the study. The participants were required to abstain from alcohol the day before the study day, on study day, and the day after the study day during each study phase. The consumption of caffeine‐containing beverages was forbidden from 10 a.m. on the day before the study day until 21.30 p.m. on the study day. Participants were required to fast overnight before the study days.

The Ethics Committee 1 of the Helsinki and Uusimaa Hospital District (record number HUS/3225/2021; November 24, 2021) and the Finnish Medicines Agency (EudraCT number 2021–003197‐31; February 9, 2022) gave approval for this study (conducted March 2022 to June 2022).

### Study participants

Twelve healthy volunteers were enrolled after giving written informed consent. The participants did not take any continuous systemic medications (including hormonal contraception), were non‐smokers, and had no clinically significant abnormalities in clinical examination or laboratory tests (hemoglobin, erythrocyte, leukocyte, and thrombocyte counts, alanine transaminase, alkaline phosphatase, gamma‐glutamyl transferase, creatinine, potassium, and sodium levels, blood glucose value, and creatine kinase). All female participants had a negative pregnancy test before the study.

### Analytical methods

Determination of plasma drug and biomarker concentrations was achieved using a Sciex 6500 Qtrap (AbSciex, Toronto, Ontario, Canada) or a Sciex 5500 Qtrap (AbSciex) liquid chromatography‐mass spectrometry (LC–MS) system. An electrospray ion source was used in all measurements. Analytes, mass transitions used for quantification, ionization mode, limits of quantification, internal standards, and reference publications for the methods are given in **Table** **S1**. The between‐day precision (CV%) and mean accuracy were below 15% and within ±15%, respectively, for all analytes at relevant concentrations.

### Genotyping

Genomic DNA was extracted from buffy coats prepared from EDTA‐anticoagulated blood samples using the Maxwell 16 LEV Blood DNA Kit on a Maxwell 16 Research automated nucleic acid extraction system (Promega, Madison, WI). The samples were genotyped with an accredited clinical pharmacogenetic panel test available at the Genome Unit of the HUS Diagnostic Center (Helsinki University Hospital, Helsinki, Finland). Genotyping was carried out with massive parallel sequencing on the Ion GeneStudio™ S5 Prime System (Thermo Fisher Scientific, Waltham, MA) as described previously.^14^ The panel covers clinically relevant variants in the *ABCG2*, *CYP2B6*, *CYP2C9*, *CYP2C19*, *CYP2D6*, *CYP3A5*, *CYP4F2*, *DPYD*, *NUDT15*, *SLCO1B1*, *TPMT*, and *VKORC1* genes. The genotypes were translated to phenotypes using standard methods.^14^

### Pharmacokinetics

Pharmacokinetic values, including peak plasma concentration (*C*_max_), time to *C*_max_ (*T*_max_), elimination half‐life (t_1/2_), and area under the concentration–time curve up to 4, 12, 23 hours, and infinity (AUC_0–4h_, AUC_0–12h_, AUC_0–23h_, and AUC_0–∞_ respectively) were calculated with Phoenix WinNonlin, Version 8.3 (Certara, Princeton, NJ) using standard non‐compartmental methods for the index drugs and their metabolites. Additionally, metabolic ratios (*C*_metabolite_/*C*_index drug_) at 2‐hour and 4‐hour timepoints and respective metabolite/index drug AUC_0–4h_, AUC_0–12h_, AUC_0–23h_, and AUC_0–∞_ ratios were calculated. As the extrapolated part of the AUC_0–∞_ exceeded 10% for some index drugs and metabolites, AUC_0–23h_ was used as the main pharmacokinetic metric for the index drugs and metabolites (**Table** **S2**). The only exception was the use of AUC_0–12h_ for caffeine and paraxanthine as caffeine consumption was allowed after collection of the 12‐hour sample. AUC_0–4h_ values for GCDCA‐3G, GCDCA‐3S, M430, M444, and solanidine were calculated with the trapezoidal rule in Excel (Microsoft, Redmond, WA). Additionally, a morning (0‐hour, C_0_) metabolic ratio and AUC_0–4h_ ratios were calculated for M430/solanidine and M444/solanidine.

### Statistical analyses

The sample size of 12 participants was estimated to be sufficient to detect an at least 30% decrease or 43% increase in the AUC of repaglinide and simvastatin, and the metabolite/parent AUC ratios of the index drugs between the grapefruit juice or lingonberry powder phase and the control phase with a power of at least 80% (α level 5%). All other variables, including the biomarker variables, were secondary outcome measures and not considered in the sample size calculations.

Individuals with poor metabolizer phenotype were excluded from statistical comparisons regarding the index drug of the respective CYP enzyme. As for GCDCA‐3G, individuals with poor function phenotype of OATP1B1 were excluded from statistical analyses. Additionally, to keep comparisons balanced, subjects lacking pharmacokinetic variable data in any study phase were excluded from the respective analyses. Prior to statistical analyses with IBM SPSS Statistics Version 29.0 for Windows (IBM Corporation, Armonk, NY), all pharmacokinetic variable values, except *T*_max_, were logarithmically transformed. Pharmacokinetic variables except *T*_max_ were compared between different study phases by repeated‐measures analysis of variance with study phase as a within‐subjects factor. *T*_max_ values were compared with the Wilcoxon signed‐rank test. Differences with *P* < 0.05 after the Bonferroni correction were considered statistically significant. The data are presented as geometric mean values or geometric mean ratios with geometric CV or 90% confidence intervals (CI) unless otherwise indicated. The statistical criterion for concluding lack of drug interaction effect in the study was that the 90% CI for the relative change in the metabolite to parent AUC ratio or index drug AUC is within 70–143% of control.

## RESULTS

### Participants

Eleven healthy volunteers (6 females and 5 males) completed all study phases. The twelfth participant withdrew after one study phase due to personal reasons and was excluded from the analyses. The mean age (± standard deviation) of study participants was 24 ± 4 years, mean height 1.73 ± 0.11 m, mean weight 66.4 ± 9.4 kg, and mean body mass index 22.1 ± 1.1 kg/m^2^. The genotypes and predicted phenotypes of relevant drug‐metabolizing enzymes and transporters are described in **Table** **S3**.

### Effect of grapefruit

**Figure 2:**
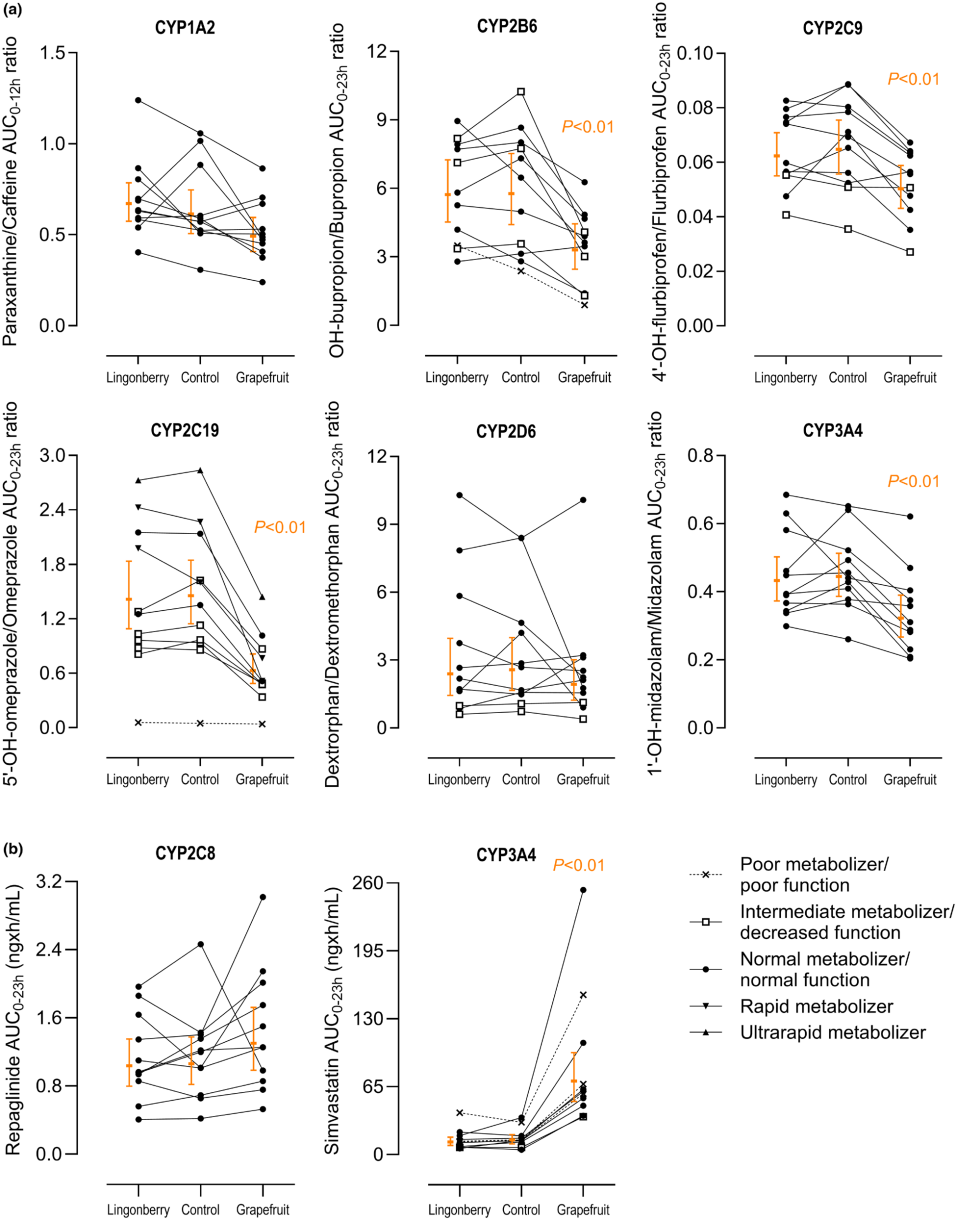
(**a**) Individual AUC_0–12h_ and AUC_0–23h_ ratios of the index drugs (caffeine/CYP1A2, bupropion/CYP2B6, flurbiprofen/CYP2C9, omeprazole/CYP2C19, dextromethorphan/CYP2D6, and midazolam/CYP3A4) and (**b**) the index drugs repaglinide (CYP2C8) and simvastatin (CYP3A4) in 11 healthy subjects with either water (control), lingonberry powder, or grapefruit juice pretreatment followed by the index drug cocktail. In all subfigures, individuals are represented with symbols for different phenotypes of CYP activities and transporter function. Geometric mean values ±90% confidence intervals are shown for the 10 or 11 individuals that did not have the respective poor metabolizer phenotype and are expressed as orange horizontal lines with error bars.

**Figure 3:**
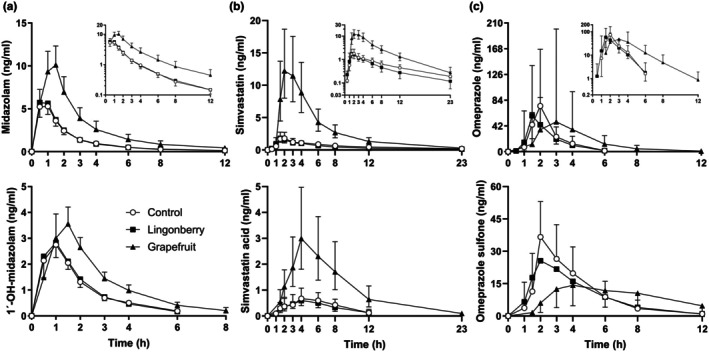
Concentrations of the CYP3A4 index drugs and their metabolites (**a**: midazolam and 1′‐hydroxymidazolam, **b**: simvastatin and simvastatin acid, and **c**: omeprazole and omeprazole sulfone) in 11 healthy subjects as geometric mean values ±90% confidence intervals in the three study phases with either water (control), lingonberry powder, or grapefruit juice pretreatment followed by the index drug cocktail.

As expected, grapefruit juice pretreatment increased the AUC_0–23h_ of the CYP3A4 index drug midazolam 2.4‐fold (90% CI: 2.2–2.6) and decreased the 1′‐hydroxymidazolam/midazolam AUC_0–23h_ ratio to 0.72‐fold (90% CI: 0.63–0.84) of control (*P <* 0.01) (**Figures** **2** **and** **3**, **Table** **1**, **Tables** **S2** **and** **S4**). In addition, the AUC_0–23h_ values of simvastatin and simvastatin acid were increased 5.0‐fold (90% CI: 4.3–5.9) and 4.1‐fold (90% CI: 3.0–5.7) after grapefruit juice pretreatment, in relation to water, respectively (*P* < 0.01) (**Figures** **2** **and** **3**, **Table** **1**, **Table** **S2**). The AUC_0–23h_ ratio of omeprazole sulfone/omeprazole, a third CYP3A4 index, was decreased to 0.60‐fold (90% CI: 0.47–0.76, *P* < 0.01) of control after grapefruit juice pretreatment (**Figure** **3**, **Table** **S4**).

**Table 1:** The AUC_0–12h_ and AUC_0–23h_ values of the index drugs and the respective AUC ratios (AUC of CYP‐selective metabolite/AUC of index drug) in healthy subjects in the three study phases (I water (control), II lingonberry powder, and III grapefruit juice pretreatment followed by the full cocktail) as geometric mean values (with geometric CV). Geometric mean ratios, GMR, (with 90% confidence intervals) compared with the control phase are indicated on the rows below each pharmacokinetic variable.

	Water (control)	Lingonberry powder	Grapefruit juice
CYP1A2 (*N* = 11)			
Caffeine AUC_0–12h_ (ng × h/mL)	9,160 (37.5%)	9,160 (37.7%)	10,900 (43.0%)
*GMR*	*Control*	1.0 (0.80–1.25)	1.19 (1.05–1.35)
*P*‐value		>0.99	0.023
Paraxanthine/caffeine AUC_0–12h_ ratio	0.61 (36.7%)	0.67 (29.3%)	0.49 (35.5%)
*GMR*	*Control*	1.09 (0.89–1.34)	0.80 (0.66–0.98)
*P*‐value		0.72	0.068
CYP2B6 (*N* = 10)^a^			
Bupropion AUC_0–23h_ (ng × h/mL)	75.3 (33.5%)	79.4 (30.6%)	116 (39.5%)
*GMR*	*Control*	1.05 (0.95–1.17)	1.54 (1.29–1.84)
*P*‐value		0.58	8.2 × 10^−4^
OH‐bupropion/bupropion AUC_0–23h_ ratio	5.77 (48.5%)	5.73 (42.2%)	3.31 (54.7%)
*GMR*	*Control*	0.99 (0.85–1.16)	0.57 (0.45–0.74)
*P*‐value		>0.99	0.0016
CYP2C8 (*N* = 11)	1.06 (50.2%)	1.04 (51.2%)	1.30 (54.7%)
Repaglinide AUC_0–23h_ (ng × h/mL)	*Control*	0.98 (0.82–1.17)	1.23 (0.91–1.65)
*GMR*		>0.99	0.31
*P*‐value			
CYP2C9 (*N* = 11)^b^			
Flurbiprofen AUC_0–23h_ (ng × h/mL)	5,970 (34.6%)	6,040 (28.4%)	6,310 (33.0%)
*GMR*	*Control*	1.01 (0.89–1.15)	1.06 (0.89–1.26)
*P*‐value		>0.99	>0.99
4′‐OH‐flurbiprofen/flurbiprofen AUC_0–23h_ ratio	0.065 (28.4%)	0.062 (23.5%)	0.050 (29.1%)
*GMR*	*Control*	0.96 (0.86–1.07)	0.78 (0.69–0.87)
*P*‐value		0.92	0.0013
CYP2C19 (*N* = 10)^c^			
Omeprazole AUC_0–23h_ (ng × h/mL)	183 (72.8%)	188 (73.2%)	366 (90.8%)
*GMR*	*Control*	1.03 (0.83–1.28)	2.00 (1.16–3.45)
*P*‐value		>0.99	0.036
5′‐OH‐omeprazole/omeprazole AUC_0–23h_ ratio	1.45 (43.0%)	1.41 (47.3%)	0.63 (46.2%)
*GMR*	*Control*	0.97 (0.89–1.06)	0.43 (0.36–0.52)
*P*‐value		>0.99	8.4 × 10^−6^
CYP2D6 (*N* = 11)^d^			
Dextromethorphan AUC_0–23h_ (ng × h/mL)	4.56 (128%)	4.85 (121%)	8.49 (95.9%)
*GMR*	*Control*	1.07 (0.83–1.36)	1.86 (1.09–3.18)
*P*‐value		>0.99	0.053
Dextrorphan/dextromethorphan AUC_0–23h_ ratio	2.57 (95.5%)	2.39 (116%)	1.92 (98.0%)
*GMR*	*Control*	0.93 (0.71–1.21)	0.75 (0.46–1.23)
*P*‐value		>0.99	0.44
CYP3A4 (*N* = 11)			
Midazolam AUC_0–23h_ (ng × h/mL)	14.8 (41.1%)	15.3 (31.7%)	35.3 (45.1%)
*GMR*	*Control*	1.04 (0.94–1.14)	2.38 (2.16–2.63)
*P*‐value		0.92	5.5 × 10^−9^
1′‐OH‐midazolam/midazolam AUC_0–23h_ ratio	0.45 (26.5%)	0.43 (27.8%)	0.32 (35.7%)
*GMR*	*Control*	0.97 (0.85–1.11)	0.72 (0.63–0.84)
*P*‐value		>0.99	0.0012
CYP3A4 (*N* = 11)^e^			
Simvastatin AUC_0–23h_ (ng × h/mL)	14.1 (62.1%)	12.2 (63.0%)	70.4 (65.5%)
*GMR*	*Control*	0.87 (0.67–1.12)	5.00 (4.25–5.88)
*P*‐value		0.49	1.6 × 10^−9^
Simvastatin acid AUC_0–23h_ (ng × h/mL)	5.52 (123%)	5.16 (127%)	22.6 (108%)
*GMR*	*Control*	0.94 (0.72–1.22)	4.10 (2.95–5.70)
*P*‐value		>0.99	4.8 × 10^−6^

^a^ CYP2B6 phenotypes were the following: 7 normal, 3 intermediate, and 1 poor metabolizer, who was excluded from the statistical analyses regarding bupropion and hydroxybupropion.

^b^ CYP2C9 phenotypes were the following: 9 normal and 2 intermediate metabolizers.

^c^ CYP2C19 phenotypes were the following: 1 ultrarapid, 2 rapid, 2 normal, 5 intermediate, and 1 poor metabolizer, who was excluded from the statistical analyses regarding omeprazole and 5′‐hydroxyomeprazole.

^d^ CYP2D6 phenotypes were the following: 9 normal and 2 intermediate metabolizers.

^e^ OATP1B1 phenotypes were the following: 7 normal, 1 decreased, and 3 poor function phenotypes. All phenotypes were included in statistical analyses.

**Figure 4:**
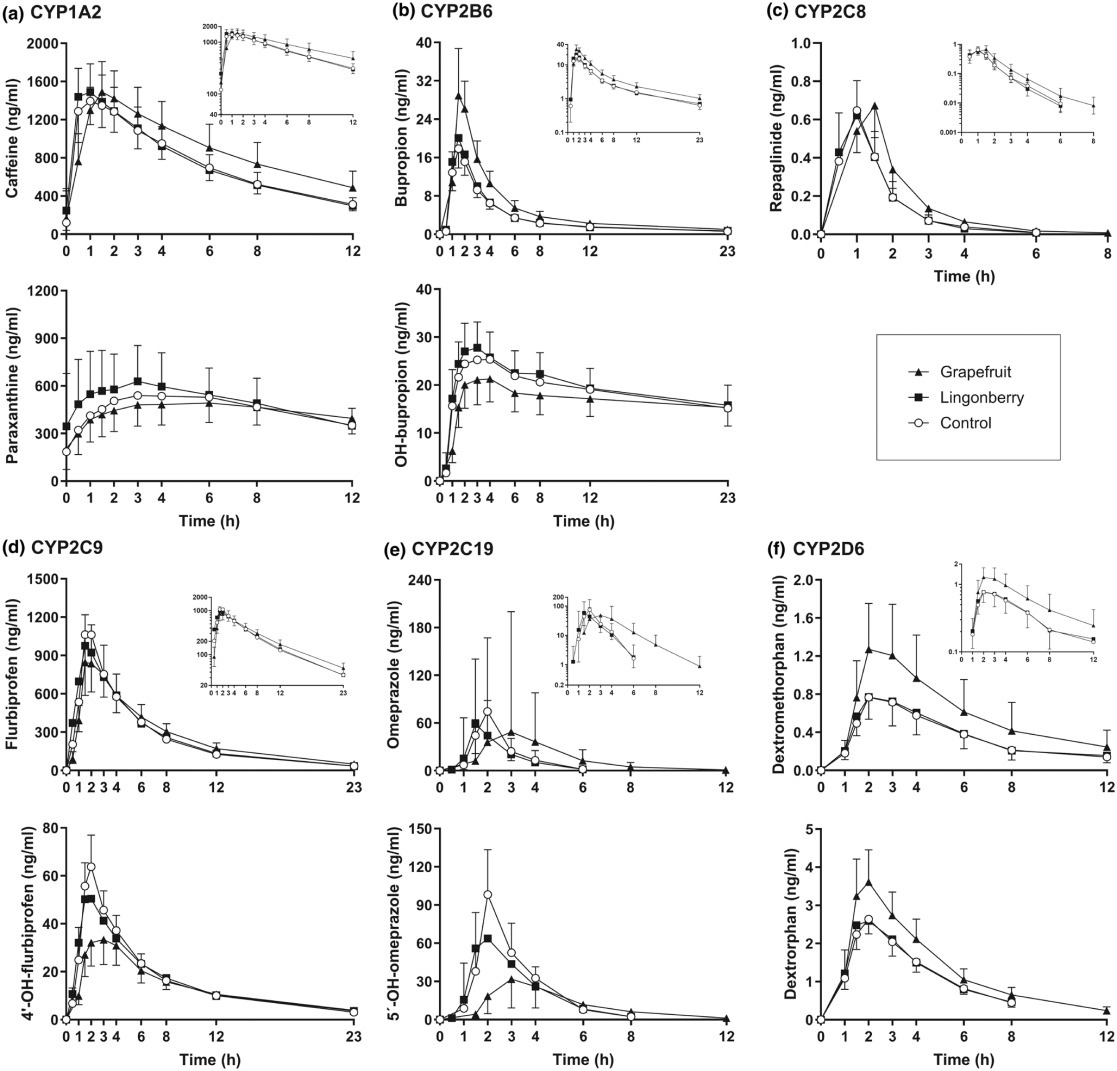
Concentrations of the index drugs and their metabolites for six CYP enzymes (**a**: CYP1A2, **b**: CYP2B6, **c**: CYP2C8, **d**: CYP2C9, **e**: CYP2C19, and **f**: CYP2D6) as geometric mean values ±90% confidence intervals in the three study phases with either water (control), lingonberry powder, or grapefruit juice pretreatment followed by the full cocktail. The data in each graph are from the 10 or 11 healthy subjects who did not have the poor metabolizer phenotype of the respective enzyme.

In addition to CYP3A4 indices, grapefruit juice decreased the hydroxybupropion/bupropion (CYP2B6), 4′‐hydroxyflurbiprofen/flurbiprofen (CYP2C9), and 5′‐hydroxyomeprazole/omeprazole (CYP2C19) AUC_0–23h_ ratios to 0.57‐fold (90% CI: 0.45–0.74), 0.78‐fold (90% CI: 0.69–0.87), and 0.43‐fold (90% CI: 0.36–0.52) of control, respectively (*P* < 0.01) (**Figure** **2**, **Table** **1**, **Table** **S4**). The AUC_0–23h_ values of bupropion and omeprazole were increased 1.54‐fold (*P* < 0.01) and 2.00‐fold (*P* = 0.036), while the AUC_0–23h_ of flurbiprofen was not significantly affected by grapefruit juice (**Figure** **4**, **Table** **1**, **Table** **S2**). Notably, the t_1/2_ of omeprazole and its two metabolites, 5′‐hydroxyomeprazole and omeprazole sulfone, were increased (*P <* 0.01) (**Figures** **3** and **4**, **Table** **S2**).

In contrast to the above findings, grapefruit juice did not significantly affect the paraxanthine/caffeine AUC_0–12h_ ratio (CYP1A2), AUC_0–23h_ of repaglinide (CYP2C8), or dextrorphan/dextromethorphan AUC_0–23h_ ratio (CYP2D6) (**Figure** **2**, **Table** **1**, **Tables** **S2** and **S4**). However, both dextromethorphan and dextrorphan *C*_max_ were increased 1.70‐fold (*P =* 0.033) and 1.38‐fold (*P* < 0.01) after grapefruit juice pretreatment, respectively (**Figure** **4**, **Table** **S2**). The biomarker measures showed that the 0‐hour metabolic ratios and AUC_0–4h_ ratios of the CYP2D6 indices M430/solanidine and M444/solanidine were not affected significantly by grapefruit juice (**Table** **S5**). Similarly, GCDCA‐3G and GCDCA‐3S C_0_ and AUC_0–4h_ values were not affected significantly by grapefruit juice (**Table** **2**).

**Table 2:** The C_0_ concentrations and AUC_0–4h_ values (based on concentrations from 8.00 a.m. to 12 a.m. on study days) of the OATP1B1 and OATP1B3 biomarkers GCDCA‐3G and GCDCA‐3S, respectively, in healthy subjects during the water (control), lingonberry powder and grapefruit juice phases expressed as geometric mean values (with geometric CV). Geometric mean ratios (GMR), and their 90% confidence intervals, compared with the control phase are indicated on the rows below each variable.

	Water (control)	Lingonberry powder	Grapefruit juice
OATP1B1 normal/decreased function (*N* = 8)^a^			
GCDCA‐3G C_0_ (ng/mL)	32.4 (64.9%)	35.1 (63.2%)	44.7 (89.8%)
*GMR*	*Control*	1.08 (0.60–1.95)	1.38 (0.60–3.17)
*P*‐value		>0.99	0.79
GCDCA‐3G AUC_0–4h_ (ng × h/mL)	124 (58.2%)	133 (56.5%)	157 (76.5%)
*GMR*	*Control*	1.07 (0.69–1.67)	1.27 (0.67–2.43)
*P*‐value		>0.99	0.81
OATP1B3 (*N* = 11)			
GCDCA‐3S C_0_ (ng/mL)	63.2 (59.4%)	71.2 (81.8%)	95.1 (49.7%)
*GMR*	*Control*	1.13 (0.72–1.77)	1.51 (1.05–2.16)
*P*‐value		>0.99	0.06
GCDCA‐3S AUC_0–4h_ (ng × h/mL)	235 (44.9%)	277 (43.0%)	305 (38.4%)
*GMR*	*Control*	1.18 (0.87–1.59)	1.30 (0.98–1.71)
*P*‐value		0.51	0.12

^a^ Individuals with poor OATP1B1 function phenotypes were excluded from statistical analyses.

### Effect of lingonberry

Compared with water pretreatment, lingonberry powder did not have any statistically significant effects on 2‐hour and 4‐hour metabolic ratios and metabolite/index drug AUC ratios of CYP indices (**Figure** **2**, **Figure** **S1**, **Table** **1**, **Table** **S4**). Neither were the parent drug pharmacokinetic variables affected by lingonberry powder pretreatment (**Figures** **3** and **4**, **Table** **1**, **Table** **S2**). GCDCA‐3G and GCDCA‐3S C_0_ and AUC_0–4h_ values were unchanged after lingonberry powder pretreatment (**Table** **2**). Lingonberry powder had no effect on solanidine metabolic ratios (**Table** **S6**).

### Safety

No adverse effects were reported during the study. The mean blood glucose values were above 4.0 mmol/L at both 2‐hour and 4‐hour timepoints in all three study phases. The lowest measured blood glucose value was 3.3 mmol/L in one participant at the 4‐hour sampling timepoint in the control phase with no symptoms of hypoglycemia. Additional carbohydrates were not required during the study.

## DISCUSSION

Our phenotyping cocktail accurately captured the well‐established inhibitory effects of grapefruit juice on the CYP3A4 index drugs midazolam and simvastatin. Additionally, grapefruit juice decreased the CYP2B6, CYP2C9, and CYP2C19 activities, indicating that the clinical CYP inhibitory effects of grapefruit juice extend beyond CYP3A4. On the contrary, lingonberry powder had no effect on CYP activities. Moreover, neither grapefruit juice nor lingonberry powder markedly affected the OATP1B1 and OATP1B3 biomarkers, indicating a lack of clinically relevant effects on these transporters.

The most important new findings in the present study were that based on respective metabolite/parent ratios, grapefruit juice can cause almost 50% inhibition of CYP2B6 and more than 50% inhibition of CYP2C19. Interestingly, these effects were greater than the effects of grapefruit juice on the CYP3A4 index based on midazolam hydroxylation. Although CYP2C19 protein is expressed in small intestinal enterocytes, CYP2B6 is predominantly expressed in the liver.^15^ Thus, it appears that the responsible grapefruit‐derived compounds are absorbed into the systemic circulation and exert their effect mainly in the liver. Bergamottin, a grapefruit juice furanocoumarin that is easily absorbed in the intestine, is a strong mechanism‐based inhibitor of CYP2B6 *in vitro*.^16^, ^17^ Moreover, the flavonoid naringenin and furanocoumarins bergamottin and dihydroxybergamottin have inhibited CYP2C19.^17^, ^18^, ^19^, ^20^

There are several observed grapefruit juice–drug interactions, where these previously unrecognized grapefruit juice effects may play an important role. For example, grapefruit juice pretreatment has increased sertraline concentrations ~ 1.5‐ to 2‐fold.^6^, ^7^ CYP2B6 and CYP2C19 are the major enzymes metabolizing sertraline while CYP3A4 plays a smaller part.^21^, ^22^ Accordingly, the grapefruit juice–sertraline interaction is better explained by inhibition of CYP2B6, CYP2C19, and CYP3A4 than inhibition of CYP3A4 alone. Another example is esketamine: grapefruit juice has increased its AUC 3‐fold, that is, more than the strong CYP3A4 inhibitors itraconazole and clarithromycin have.^23^, ^24^, ^25^ As esketamine is metabolized to a significant extent by CYP2B6,^25^ our results suggest that the effect of grapefruit juice on esketamine AUC is partially explained by inhibition of CYP2B6. A third and perhaps the most striking example is the effect of grapefruit juice on clopidogrel, as grapefruit juice pretreatment reduced the concentrations of the active thiol metabolite of clopidogrel to only about 14% of control, resulting in decreased platelet‐inhibitory effect.^4^ While the role of CYP3A4 in the two‐step conversion of clopidogrel to the thiol metabolite is relatively small, CYP2B6 and CYP2C19 are significantly involved.^5^, ^26^ Again, the grapefruit juice–clopidogrel interaction is best explained by inhibition of all these three enzymes.

CYP2C9‐mediated flurbiprofen hydroxylation was slightly inhibited by grapefruit juice. In some clinical trials, grapefruit juice pretreatment has not affected the pharmacokinetics of the CYP2C9 substrates glibenclamide (AUC) and tolbutamide (metabolite levels) significantly.^27^, ^28^ However, in another clinical trial with a subtherapeutic dose of glibenclamide, grapefruit juice decreased the concentrations of glibenclamide, which was explained by OATP2B1 inhibition.^29^ It is possible that these studies did not capture the relatively weak CYP2C9 inhibitory effect because the studied substrate drugs were not as sensitive as the hydroxyflurbiprofen/flurbiprofen metabolic ratio and because the OATP2B1 inhibitory effect counteracted the CYP2C9 inhibitory effect. *In vitro*, grapefruit juice flavonoids and furanocoumarins have inhibited CYP2C9.^16^, ^17^, ^18^, ^19^, ^30^ It is likely that the inhibition of flurbiprofen metabolism primarily took place in the liver, as CYP2C9 is expressed in the intestinal wall less abundantly than CYP3A4.^15^, ^31^ Taken together, grapefruit juice can cause weak inhibition of CYP2C9 that may be clinically relevant with sensitive CYP2C9 substrates with a narrow therapeutic index, such as warfarin.

The observed statistically nonsignificant 20% decrease in paraxanthine/caffeine AUC_0–12h_ ratio after grapefruit juice pretreatment in our study is in line with existing literature. In previous clinical trials, grapefruit juice has affected caffeine pharmacokinetics consistent with weak (up to 28%) CYP1A2 inhibition, although the changes have not always reached statistical significance.^27^, ^32^, ^33^ Of note, caffeine AUC_0–12h_ was increased 1.19‐fold by grapefruit juice in our study. However, this variable is not as reliable as the paraxanthine to caffeine ratio because some participants had residuals of caffeine in their blood in the mornings of the study days. Additionally, grapefruit juice has had no effect on the pharmacokinetics of another CYP1A2 substrate, clozapine.^34^ *In vitro*, grapefruit juice ingredients have inhibited CYP1A2 modestly or weakly only^18^, ^32^, ^35^ and CYP1A2 is not expressed in the small intestine.^15^, ^31^ In summary, grapefruit juice may cause weak inhibition of CYP1A2, but this effect is hardly of clinical relevance.

Based on our findings, grapefruit juice does not inhibit CYP2C8 or CYP2D6. We observed a 20% increase in the AUC_0–23h_ of repaglinide, which was not statistically significant, although an increase was observed in 10 of the 11 subjects. This finding is well in line with a previous clinical trial where repaglinide AUC was increased 1.13‐fold after grapefruit juice ingestion; the increase in repaglinide AUC can be attributed to inhibition of intestinal CYP3A4 by grapefruit since repaglinide is metabolized to a small extent by CYP3A4.^36^ In previous studies, grapefruit juice has not affected the CYP2D6‐mediated dextromethorphan or sparteine metabolism.^27^, ^37^, ^38^, ^39^ In our study, the concentrations of both dextromethorphan and dextrorphan were increased by about 40–70%, similarly to a previous study where this was explained by decreased CYP3A4‐mediated first‐pass metabolism of dextromethorphan in the gut wall.^37^ In our study, the lack of CYP2D6 inhibition was corroborated by the unaffected CYP2D6 index solanidine metabolic ratios.^40^ *In vitro*, the inhibitory effects of bergamottin, 6’,7’‐dihydroxybergamottin, and naringin have usually been weaker on CYP2D6 than on other CYP enzymes.^16^, ^17^, ^18^, ^19^, ^30^

Clinically, grapefruit juice is a well‐established inhibitor of CYP3A4 and markedly increases the concentrations of multiple CYP3A4 substrates.^1^, ^27^, ^41^ *In vitro*, CYP3A4 inhibition has been observed for grapefruit juice constituents including flavonoids (naringin, naringenin) and furanocoumarins (bergamottin and 6′,7′‐dihydroxybergamottin).^1^, ^17^, ^18^, ^19^ Mechanism‐based inactivation of CYP3A4 by 6′,7′‐dihydroxybergamottin is generally considered the main mechanism of clinical CYP3A4 inhibition. CYP3A4 is the major CYP enzyme in the intestinal wall, and continuous grapefruit juice consumption has markedly decreased CYP3A4 protein abundance in the small intestinal wall.^2^

In our study, the CYP3A4 substrates simvastatin and midazolam acted as positive controls for the grapefruit juice effects. Our findings of ~ 5‐fold increased AUC_0–23h_ values of simvastatin and simvastatin acid after grapefruit juice pretreatment are well in line with expectations. In previous studies, the AUC values of simvastatin and simvastatin acid have increased 4‐fold to 16‐fold and 3‐fold to 9‐fold, respectively, and the largest AUC increases have been observed with repeated dosing of double‐strength grapefruit juice.^11^, ^12^, ^13^, ^42^ In our study, the AUC_0–23h_ of midazolam was increased 2.4‐fold, which corresponds well with previous clinical trials, where grapefruit juice regimens typically increased midazolam AUC 1.5‐ to 1.7‐fold.^43^, ^44^, ^45^ Of note, multiple doses of grapefruit juice have prolonged the t_1/2_ of triazolam and midazolam and decreased erythromycin breath‐test results, indicating that continuous grapefruit juice consumption can affect CYP3A4 activity also in the liver.^41^, ^46^ Accordingly, it appears that furanocoumarins, the main mediators of CYP3A4 inhibition,^41^, ^47^ can reach the liver in sufficient concentrations.

Grapefruit juice did not affect the OATP1B1 or OATP1B3 indices statistically significantly, although grapefruit juice and its ingredients have inhibited these transporters *in vitro*.^35^, ^48^, ^49^ Grapefruit juice has increased the AUC values of both pitavastatin acid and pitavastatin lactone by 14%, which was thought to suggest slight inhibition of OATP1B1 by grapefruit juice.^50^ On the other hand, the bioavailability of pravastatin, which is also transported by OATP1B1, is not affected by grapefruit juice.^3^ Overall, the lack of significant effect of grapefruit juice on sensitive OATP1B1 and OATP1B3 biomarkers in our study suggests that grapefruit juice consumption has no clinically relevant inhibitory effects on these OATP transporters, although due to the relatively wide confidence intervals, slight inhibition cannot be excluded.

Continuous lingonberry powder intake three times per day for 9 days had no effect on the activity of any of the seven CYP enzymes, OATP1B1, or OATP1B3. This contrasts with the findings in mice, where repeated lingonberry powder intake increased the mRNA expression of several CYP enzymes.^9^ The current dosage of 5 g lingonberry powder three times per day corresponds to as much as 2 dL fresh lingonberries per day, and the pretreatment lasted 9 days in total, with the last dose taken 12–15 hr before cocktail administration to avoid simultaneous inhibitory effect on CYP enzymes. Thus, any clinically significant induction should have developed during the pretreatment period. Of note, our study was not designed to exclude inhibition of CYP enzymes or OATP transporters by lingonberry powder. Our results highlight the differences in xenobiotic metabolizing enzyme regulation between different species and emphasize the need to obtain data from humans before making any firm clinical conclusions or recommendations.

In conclusion, repeated grapefruit juice intake causes moderate inhibition of CYP2B6 and CYP2C19 and can weakly inhibit CYP2C9, in addition to moderate–strong inhibition of CYP3A4. By contrast, sustained lingonberry powder intake is unlikely to induce CYP enzymes in humans. Moreover, neither grapefruit juice nor lingonberry powder causes clinically relevant effects on OATP1B1 or OATP1B3 activities. Importantly, the results reveal previously unrecognized CYP inhibition by grapefruit juice in humans. This new knowledge changes the interpretation of previously documented grapefruit juice–drug interactions and indicates that grapefruit juice cannot be regarded as a selective inhibitor of intestinal CYP3A4.

## FUNDING

This study was supported by grants from the Academy of Finland [Grant decision 325667, 2019], the Sigrid Jusélius Foundation (Grant numbers 8037 and 250162; Helsinki, Finland), and by State funding for university‐level health research (TYH2021304, TYH2022301, TYH2023412, and TYH2024301; Hospital District of Helsinki and Uusimaa, Finland). In addition, Aurinsalo L received personal grants from the Finnish Medical Foundation (Grant numbers: 4697, 2021, and 7649, 2024; Helsinki, Finland) and Orion Research Foundation (Grant decision in 2024; Espoo, Finland).

## CONFLICT OF INTEREST

The authors declared no competing interests for this work.

## AUTHOR CONTRIBUTIONS

L.A., M.K., M.Ne., M.Ni., A.T., and J.T.B. wrote the manuscript. L.A., E.M., M.Ni., A.T., and J.T.B. designed the research. L.A., O.L.‐R., M.K., M.Ne., M.Ni., and J.T.B. performed the research. L.A., A.T., and J.T.B. analyzed the data.

## Supporting information


Data S1.
