# Supplementary material for: Repeated Intake of Grapefruit Juice Inhibits CYP2B6, CYP2C9, CYP2C19, and CYP3A4 while Lingonberry Powder Does Not Induce Major CYP Enzymes in Humans
Source: Clin Pharmacol Ther. 2025 Dec 14;119(4):953–63. doi: 10.1002/cpt.70165 (PMC12997508; doi:10.1002/cpt.70165)
Supplement: Supplementary file 1 — Data S1. [file CPT-119-953-s001.pdf]

SUPPLEMENTARY INFORMATION FOR

Repeated intake of grapefruit juice inhibits CYP2B6, CYP2C9, CYP2C19, and CYP3A4 while lingonberry powder does not induce major CYP enzymes in humans

Aurinsalo, Laura[1,2,3]; Lapatto-Reiniluoto, Outi[1,2,3,4]; Kurkela, Mika[1,2]; Neuvonen, Mikko[1,2]; Moilanen, Eeva[5]; Niemi, Mikko[1,2,3]; Tornio, Aleksi[6,7]; Backman, Janne T[1,2,3]

[1]Department of Clinical Pharmacology, University of Helsinki, Helsinki, Finland

[2]Individualized Drug Therapy Research Program, Faculty of Medicine, University of Helsinki, Helsinki, Finland

[3]Department of Clinical Pharmacology, HUS Diagnostic Center, Helsinki University Hospital, Helsinki, Finland

[4]HUS Pharmacy, Helsinki University Hospital, Helsinki, Finland

[5]The Immunopharmacology Research Group, Faculty of Medicine and Health Technology, Tampere University, and Tampere University Hospital, Tampere, Finland

[6]Integrative Physiology and Pharmacology, Institute of Biomedicine, University of Turku, Turku, Finland

[7]Unit of Clinical Pharmacology, Turku University Hospital, Turku, Finland

TABLE OF CONTENTS……………………………………………………………..……2
SUPPLEMENTARY FIGURES…………………………………………………………….3
Figure S1……………………………………………………………………………………3
SUPPLEMENTARY TABLES…………………………………………………………...4
Table S1……………………………………………………………………………………..4
Table S2…………………………………………………………………………..…………6
Table S3……………………………………………………………………………………18
Table S4…………………………………………………………………………..……..20
Table S5…………………………………………………………………………...……..26
Table S6……………………………………………………………………………………27

SUPPLEMENTARY FIGURES

Figure S1. Individual 2-hour metabolic ratios (2h MR) of the index drugs in healthy subjects in the three study phases with either water (control), lingonberry powder or grapefruit juice pretreatment followed by the full cocktail. In all subfigures, individuals are represented with symbols for different phenotypes of CYP activities. Geometric mean values +/- 90% confidence intervals calculated for 10 or 11 individuals (poor metabolizers for given enzyme were excluded) are shown as orange horizontal lines with error bars.

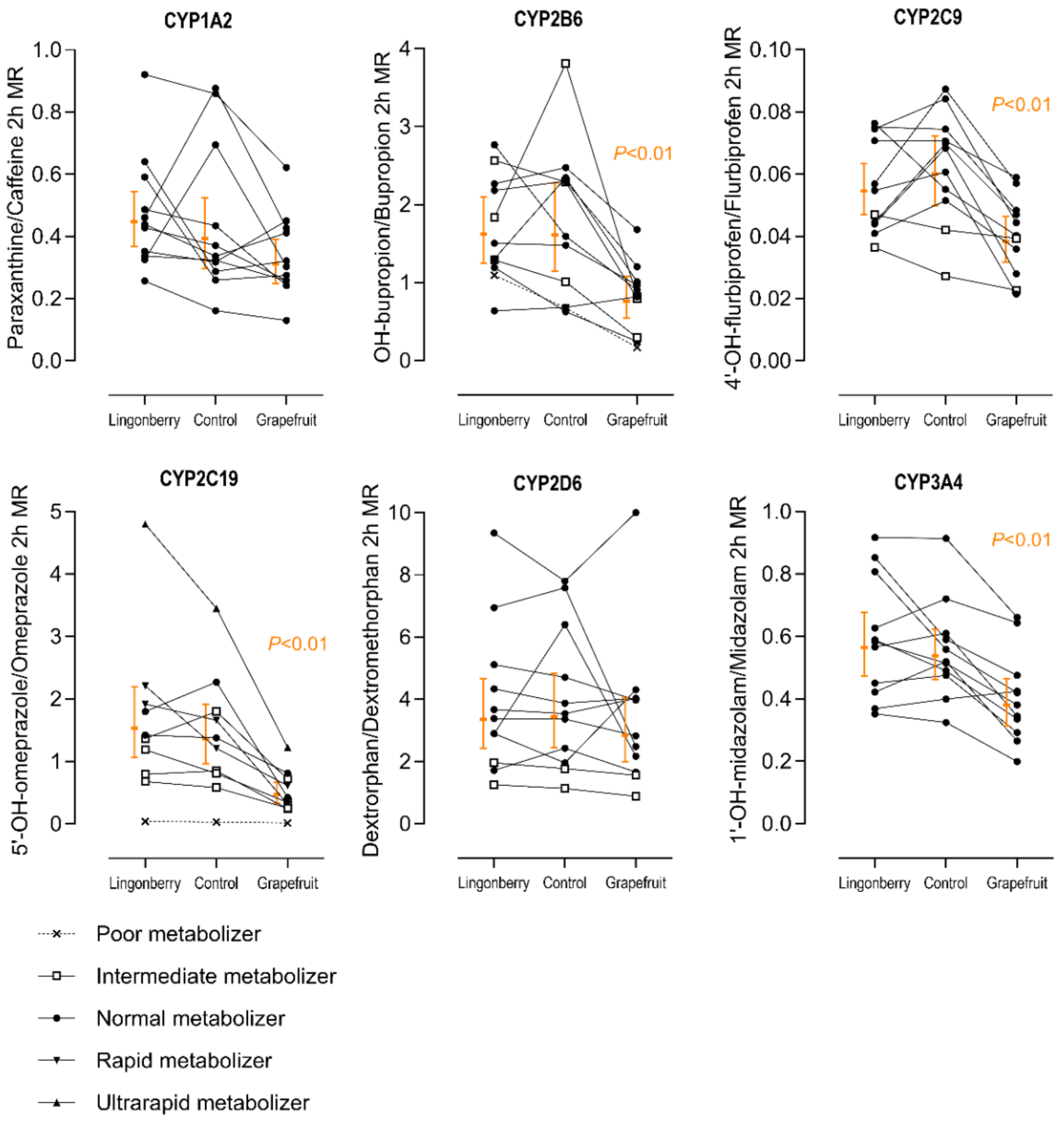

SUPPLEMENTARY TABLES

Table S1. Mass spectrometry parameters for the index drugs, biomarkers and their metabolites.

Analyte	MRM transition (*m/z* > *m/z*)	Ionization (+/-)	LOQ (ng/mL)	Internal standard	Reference
Caffeine	195 > 138	+	20	Caffeine-d9	Aurinsalo et al.
Paraxanthine	181 > 124	+	20	Paraxanthine-d3	Aurinsalo et al.
Bupropion	240 > 184	+	0.16	Bupropion-d9	Aurinsalo et al.
Hydroxybupropion	256 > 238	+	0.8	Hydroxybupropion-d6	Aurinsalo et al.
Flurbiprofen	199 > 199	-	15	Flurbiprofen-d5	Aurinsalo et al.
Hydroxyflurbiprofen	259 > 215	-	1.0	Flurbiprofen-d5	Aurinsalo et al.
Omeprazole	346 > 198	+	0.5	Omeprazole-d5	Aurinsalo et al.
5-Hydroxyomeprazole	362 > 214	+	0.3	5-Hydroxyomeprazole-d3	Aurinsalo et al.
Omeprazole sulfone	362 > 150	+	0.4	Omeprazole-d3 sulfone	Aurinsalo et al.
Dextromethorphan	272 > 215	+	0.15	Dextromethorphan-d3	Aurinsalo et al.
Dexthorphan	258 > 199	+	0.2	Dexthorphan-d3	Aurinsalo et al.
Midazolam	326 > 291	+	0.08	Midazolam-d6	Aurinsalo et al.
1-Hydroxymidazolam	342 > 324	+	0.12	1-Hydroxymidazolam-d4	Aurinsalo et al.
Simvastatin	436 > 285	+	0.1	Simvastatin-d6	Mykkänen et al.
Simvastatin acid	437 > 303	+	0.1	Simvastatin acid-d6	Mykkänen et al.
Repaglinide	453 > 230	+	0.01	Repaglinide-d5	Piha et al.
GCDCA-3G	624 > 448	-	0.5	GCDCA-3G-d4	Rago et al.
GCDCA-3S	528 > 448	-	0.5	GCDCA-3S-d5	Rago et al.
Solanidine	398 > 98	+ (IS -)	0.025	Tauroursodeoxycholate-d5	Kiiski et al.
Solanidine metabolite M444	444 > 98	+ (IS -)	S/N 10	Tauroursodeoxycholate-d5	Kiiski et al.
Solanidine metabolite M430	430 > 98	+ (IS -)	S/N 10	Tauroursodeoxycholate-d5	Kiiski et al.

GCDCA-3G, glycochenodeoxycholate 3-O-glucuronide; GCDCA-3S, glycochenodeoxycholate 3-sulfate; IS, internal standard; LOQ, limit of quantification; MRM, multiple reaction monitoring; S/N, signal-to-noise ratio

References:

Aurinsalo, L., et al., A phenotyping tool for seven cytochrome P450 enzymes and two transporters: application to examine the effects of clopidogrel and gemfibrozil. *Clin Pharmacol Ther*. **117(6)**, 1732-1742 (2025).

Kiiski, J.I., et al., Solanidine is a sensitive and specific dietary biomarker for CYP2D6 activity. *Hum. Genomics.* **18(1)**, 11 (2024).

Mykkänen, A.J.H., et al., Genomewide association study of simvastatin pharmacokinetics. *Clin Pharmacol Ther*. **112(3)**, 676-686 (2022).

Piha, M.O.W., et al., Candesartan has no clinically meaningful effect on the plasma concentrations of CYP2C8 substrate repaglinide in humans. *Drug Metab. Dispos.* **52(12)**,1388-1395 (2024).

Rago, B., Tierney, B., Rodrigues, A.D., Holliman C, L.H., and Ramanathan, R., A multiplex HRMS assay for quantifying selected human plasma bile acids as candidate OATP biomarkers. *Bioanalysis.* **10(9)**, 645-657 (2018).

Table S2. Pharmacokinetic variables of the index drugs and their metabolites in healthy subjects in the three study phases (water (control), lingonberry powder and grapefruit juice) as geometric mean values (with geometric CV) except for T_{max}, which is given as median with range. Geometric mean ratios (GMR) (+/- 90% confidence interval) compared to the control phase are indicated on the rows below each pharmacokinetic variable.

	Water (control)	Lingonberry powder	Grapefruit juice
**Caffeine (N=11)**			
C_{max} (ng/mL)	1490 (32.3%)	1540 (30.5%)	1550 (34.0%)
GMR	*Control*	1.03 (0.86-1.24)	1.04 (0.92-1.16)
P-value		>0.99	>0.99
T_{max} (h)	0.5 (0.5-1.5)	1.0 (0.5-1.5)	1.5 (1.0-2.0)
P-value	*Control*	0.79	0.022
AUC_{0-12h} (ng×h/mL)	9160 (37.5%)	9160 (37.7%)	10 900 (43.0%)
GMR	*Control*	1.00 (0.80-1.25)	1.19 (1.05-1.35)
P-value		>0.99	0.023
$AUC_{0-\infty}$ (ng×h/mL)	11 600 (40.1%)	11 400 (42.0%)	16 200 (61.0%)
GMR	*Control*	0.99 (0.78-1.25)	1.40 (1.15-1.71)
P-value		>0.99	0.0077
$t_{1/2}$ (h)	5.06 (20.6%)	4.90 (21.5%)	6.86 (35.4%)
GMR	*Control*	0.97 (0.88-1.07)	1.36 (1.13-1.64)
P-value		0.95	0.0091
**Paraxanthine (N=11)**			
C_{max} (ng/mL)	591 (77.3%)	659 (68.0%)	527 (61.0%)
GMR	*Control*	1.12 (0.72-1.72)	0.89 (0.66-1.20)
P-value		>0.99	0.82
T_{max} (h)	4.0 (0.5-6.0)	3.0 (0.5-4.0)	6.0 (0.5-12.0)
P-value	*Control*	>0.99	>0.99

**Paraxanthine (N=11)**			
AUC_{0-12h} (ng×h/mL)	5630 (68.8%)	6150 (60.1%)	5390 (62.4%)
GMR	*Control*	1.09 (0.73-1.63)	0.96 (0.73-1.25)
P-value		>0.99	>0.99
$AUC_{0-\infty}$ (ng×h/mL) (N=8)[1]	11 400 (61.7%)	10 500 (53.0%)	14 700 (73.2%)
GMR	*Control*	0.92 (0.62-1.36)	1.29 (1.08-1.54)
P-value		>0.99	0.024
$t_{1/2}$ (h) (N=8)[1]	9.32 (27.7%)	8.77 (43.6%)	15.2 (45.8%)
GMR	*Control*	0.94 (0.71-1.25)	1.63 (1.05-2.51)
P-value		>0.99	0.066
**Bupropion (N=10)[2]**			
C_{max} (ng/mL)	19.5 (44.8%)	20.8 (35.5%)	31.4 (44.8%)
GMR	*Control*	1.07 (0.91-1.26)	1.61 (1.34-1.93)
P-value		0.78	4.3×10^{-4}
T_{max} (h)	1.5 (1.0-2.0)	1.5 (1.0-2.0)	1.5 (1.5-2.0)
P-value	*Control*	>0.99	0.13
AUC_{0-23h} (ng×h/mL)	75.3 (33.5%)	79.4 (30.6%)	116 (39.5%)
GMR	*Control*	1.05 (0.95-1.17)	1.54 (1.29-1.84)
P-value		0.58	8.2×10^{-4}
$AUC_{0-\infty}$ (ng×h/mL)	82.4 (34.5%)	87.6 (31.3%)	127 (40.0%)
GMR	*Control*	1.06 (0.96-1.18)	1.54 (1.30-1.81)
P-value		0.43	4.7×10^{-4}
$t_{1/2}$ (h)	7.07 (22.8%)	7.54 (18.6%)	7.19 (11.6%)
GMR	*Control*	1.07 (0.97-1.18)	1.02 (0.87-1.19)
P-value		0.34	>0.99

**OH-bupropion (N=10)**[2]			
C_{max} (ng/mL)	26.8 (33.2%)	28.8 (33.9%)	22.9 (45.8%)
GMR	*Control*	1.07 (0.86-1.34)	0.86 (0.66-1.11)
P-value		0.95	0.41
T_{max} (h)	3.0 (2.0-4.0)	2.5 (2.0-4.0)	4.0 (2.0-23.0)
P-value	*Control*	0.14	0.18
$AUC_{0\text{-}23h}$ (ng×h/mL)	435 (27.3%)	455 (33.3%)	384 (46.5%)
GMR	*Control*	1.05 (0.90-1.21)	0.88 (0.69-1.13)
P-value		>0.99	0.57
$AUC_{0\text{-}\infty}$ (ng×h/mL) (N=9)[3]	950 (30.6%)	978 (50.7%)	1150 (67.9%)
GMR	*Control*	1.03 (0.86-1.23)	1.21 (0.87-1.69)
P-value		>0.99	0.45
$t_{1/2}$ (h) (N=9)[3]	23.9 (14.8%)	23.5 (26.3%)	36.1 (30.9%)
GMR	*Control*	0.99 (0.78-1.25)	1.51 (1.18-1.93)
P-value		>0.99	0.010
**Repaglinide (N=11)**			
C_{max} (ng/mL)	0.69 (49.9%)	0.66 (47.9%)	0.74 (41.2%)
GMR	*Control*	0.95 (0.72-1.24)	1.07 (0.80-1.42)
P-value		>0.99	>0.99
T_{max} (h)	1.0 (0.5-1.0)	1.0 (0.5-1.5)	1.5 (0.5-1.5)
P-value	*Control*	>0.99	0.21
$AUC_{0\text{-}4h}$ (ng×h/mL)	0.98 (47.0%)	0.98 (48.7%)	1.14 (48.6%)
GMR	*Control*	1.00 (0.85-1.18)	1.16 (0.89-1.52)
P-value		>0.99	0.47

**Repaglinide (N=11)**			
AUC_{0-23h} (ng×h/mL)	1.06 (50.2%)	1.04 (51.2%)	1.30 (54.7%)
GMR	*Control*	0.98 (0.82-1.17)	1.23 (0.91-1.65)
P-value		>0.99	0.31
$AUC_{0-\infty}$ (ng×h/mL)	1.06 (49.7%)	1.04 (50.8%)	1.30 (53.4%)
GMR	*Control*	0.98 (0.82-1.17)	1.22 (0.92-1.63)
P-value		>0.99	0.31
$t_{1/2}$ (h)	1.06 (44.8%)	0.92 (32.9%)	1.26 (42.0%)
GMR	*Control*	0.87 (0.68-1.11)	1.19 (0.85-1.68)
P-value		0.48	0.55
**Flurbiprofen (N=11)[4]**			
C_{max} (ng/mL)	1200 (33.8%)	1010 (38.2%)	1010 (60.0%)
GMR	*Control*	0.84 (0.66-1.07)	0.84 (0.57-1.24)
P-value		0.27	0.68
T_{max} (h)	1.5 (1.0-2.0)	1.5 (1.0-4.0)	1.5 (1.5-3.0)
P-value	*Control*	>0.99	0.21
AUC_{0-23h} (ng×h/mL)	5970 (34.6%)	6040 (28.4%)	6310 (33.0%)
GMR	*Control*	1.01 (0.89-1.15)	1.06 (0.89-1.26)
P-value		>0.99	>0.99
$AUC_{0-\infty}$ (ng×h/mL)	6240 (36.5%)	6330 (30.6%)	6770 (32.3%)
GMR	*Control*	1.01 (0.89-1.15)	1.08 (0.92-1.29)
P-value		>0.99	0.62
$t_{1/2}$ (h)	4.88 (20.4%)	5.05 (22.6%)	5.56 (21.7%)
GMR	*Control*	1.03 (0.97-1.10)	1.14 (1.01-1.28)
P-value		0.47	0.073

**4’-OH-flurbiprofen (N=11)[4]**			
C_{max} (ng/mL)	67.6 (31.7%)	53.1 (40.4%)	37.4 (64.0%)
GMR	*Control*	0.78 (0.62-0.98)	0.55 (0.41-0.75)
P-value		0.078	0.0027
T_{max} (h)	2.0 (1.0-2.0)	1.5 (1.5-2.0)	3.0 (1.5-8.0)
P-value	*Control*	>0.99	0.017
AUC_{0-23h} (ng×h/mL)	387 (23.0%)	377 (28.5%)	318 (45.6%)
GMR	*Control*	0.97 (0.89-1.07)	0.82 (0.68-0.99)
P-value		>0.99	0.075
$AUC_{0-\infty}$ (ng×h/mL)	425 (25.8%)	423 (28.6%)	369 (42.3%)
GMR	*Control*	1.00 (0.91-1.09)	0.87 (0.73-1.03)
P-value		>0.99	0.18
$t_{1/2}$ (h)	5.71 (38.6%)	6.11 (38.2%)	6.76 (38.7%)
GMR	*Control*	1.07 (0.96-1.19)	1.18 (0.97-1.44)
P-value		0.41	0.17
**Omeprazole (N=10)[5]**			
C_{max} (ng/mL)	133 (85.2%)	126 (103%)	137 (163%)
GMR	*Control*	0.95 (0.62-1.44)	1.03 (0.42-2.50)
P-value		>0.99	>0.99
T_{max} (h)	1.8 (1.0-3.0)	1.5 (0.5-6.0)	3.0 (1.5-12.0)
P-value	*Control*	>0.99	0.052
AUC_{0-23h} (ng×h/mL)	183 (72.8%)	188 (73.2%)	366 (90.8%)
GMR	*Control*	1.03 (0.83-1.28)	2.00 (1.16-3.45)
P-value		>0.99	0.036

**Omeprazole (N=10)[5]**			
$AUC_{0-\infty}$ (ng×h/mL) (N=7)[6]	193 (63.5%)	206 (59.7%)	492 (66.1%)
GMR	*Control*	1.07 (0.91-1.26)	2.55 (2.16-3.02)
P-value		0.67	$1.8×10^{-5}$
$t_{1/2}$ (h) (N=7)[6]	0.59 (17.6%)	0.61 (21.2%)	0.95 (20.0%)
GMR	*Control*	1.02 (0.91-1.15)	1.60 (1.43-1.79)
P-value		>0.99	$9.8×10^{-5}$
**5'-OH-omeprazole (N=10)[5]**			
C_{max} (ng/mL)	128 (48.1%)	122 (55.3%)	66.4 (127%)
GMR	*Control*	0.95 (0.67-1.33)	0.52 (0.25-1.06)
P-value		>0.99	0.13
T_{max} (h)	2.0 (1.0-3.0)	1.5 (1.0-6.0)	3.0 (2.0-12.0)
P-value	*Control*	0.96	0.034
AUC_{0-23h} (ng×h/mL)	266 (35.1%)	266 (29.7%)	230 (67.1%)
GMR	*Control*	1.00 (0.84-1.19)	0.87 (0.55-1.35)
P-value		>0.99	0.97
$AUC_{0-\infty}$ (ng×h/mL) (N=8)[7]	273 (25.4%)	267 (28.9%)	299 (21.2%)
GMR	*Control*	0.98 (0.79-1.21)	1.10 (0.98-1.23)
P-value		>0.99	0.21
$t_{1/2}$ (h) (N=8)[7]	1.00 (17.4%)	1.00 (10.1%)	1.29 (25.4%)
GMR	*Control*	1.00 (0.86-1.15)	1.28 (1.16-1.42)
P-value		>0.99	0.0015
**Omeprazole sulfone (N=10)[5]**			
C_{max} (ng/mL)	46.2 (61.3%)	41.4 (88.1%)	26.1 (139%)
GMR	*Control*	0.90 (0.60-1.33)	0.57 (0.30-1.05)
P-value		>0.99	0.13

**Omeprazole sulfone (N=10)[5]**			
T_{max} (h)	2.0 (1.0-3.0)	2.0 (1.0-6.0)	4.0 (2.0-12.0)
P-value	*Control*	>0.99	0.0098
AUC_{0-23h} (ng×h/mL)	144 (84.3%)	140 (93.2%)	172 (153%)
GMR	*Control*	0.97 (0.75-1.24)	1.19 (0.65-2.20)
P-value		>0.99	>0.99
$AUC_{0-\infty}$ (ng×h/mL) (N=7)[8]	147 (82.4%)	165 (72.7%)	221 (178%)
GMR	*Control*	1.12 (0.96-1.31)	1.50 (0.92-2.46)
P-value		0.23	0.18
$t_{1/2}$ (h) (N=7)[8]	1.58 (29.3%)	1.68 (27.9%)	2.65 (37.2%)
GMR	*Control*	1.06 (0.96-1.17)	1.68 (1.52-1.87)
P-value		0.34	3.7×10^{-5}
**Dextromethorphan (N=11)[9]**			
C_{max} (ng/mL)	0.818 (77.2%)	0.814 (70.3%)	1.39 (71.9%)
GMR	*Control*	1.00 (0.82-1.21)	1.70 (1.13-2.56)
P-value		>0.99	0.033
T_{max} (h)	2.0 (2.0-4.0)	2.0 (2.0-3.0)	2.0 (2.0-3.0)
P-value	*Control*	0.73	>0.99
AUC_{0-23h} (ng×h/mL)	4.56 (128%)	4.85 (121%)	8.49 (95.9%)
GMR	*Control*	1.07 (0.83-1.36)	1.86 (1.09-3.18)
P-value		>0.99	0.053
$AUC_{0-\infty}$ (ng×h/mL) (N=10)[10]	6.30 (82.0%)	6.90 (81.3%)	9.63 (95.7%)
GMR	*Control*	1.10 (0.83-1.45)	1.53 (0.98-2.39)
P-value		0.94	0.12

**Dextromethorphan (N=11)**[9]			
$t_{1/2}$ (h) (N=10)[10]	4.26 (20.9%)	4.98 (25.7%)	4.54 (27.1%)
GMR	*Control*	1.17 (0.96-1.43)	1.07 (0.91-1.25)
P-value		0.22	0.77
**Dextrorphan (N=11)**[9]			
C_{max} (ng/mL)	2.69 (31.1%)	2.76 (35.7%)	3.72 (41.2%)
GMR	*Control*	1.03 (0.89-1.18)	1.38 (1.18-1.62)
P-value		>0.99	0.0020
T_{max} (h)	2.0 (1.5-2.0)	2.0 (1.5-3.0)	2.0 (1.5-3.0)
P-value	*Control*	>0.99	>0.99
AUC_{0-23h} (ng×h/mL)	11.7 (38.9%)	11.6 (40.2%)	16.3 (44.8%)
GMR	*Control*	0.99 (0.85-1.15)	1.40 (1.22-1.59)
P-value		>0.99	4.7×10^{-4}
$AUC_{0-\infty}$ (ng×h/mL)	12.2 (34.9%)	12.2 (36.2%)	16.4 (41.7%)
GMR	*Control*	1.00 (0.88-1.14)	1.35 (1.19-1.53)
P-value		>0.99	6.3×10^{-4}
$t_{1/2}$ (h)	2.54 (15.3%)	2.46 (13.4%)	2.50 (18.8%)
GMR	*Control*	0.97 (0.91-1.04)	0.99 (0.91-1.07)
P-value		0.63	>0.99
**Midazolam (N=11)**			
C_{max} (ng/mL)	6.08 (34.9%)	6.37 (33.0%)	11.1 (30.3%)
GMR	*Control*	1.05 (0.91-1.21)	1.82 (1.64-2.01)
P-value		0.96	2.6×10^{-7}
T_{max} (h)	0.5 (0.5-1.0)	0.5 (0.5-1.0)	1.5 (1.0-1.5)
P-value	*Control*	>0.99	0.0091

**Midazolam (N=11)**			
$AUC_{0\text{-}23h}$ (ng×h/mL)	14.8 (41.1%)	15.3 (31.7%)	35.3 (45.1%)
GMR	*Control*	1.04 (0.94-1.14)	2.38 (2.16-2.63)
P-value		0.92	5.5×10^{-9}
$AUC_{0\text{-}\infty}$ (ng×h/mL)	14.7 (42.5%)	15.3 (32.2%)	35.4 (47.4%)
GMR	*Control*	1.04 (0.94-1.15)	2.41 (2.17-2.68)
P-value		0.81	8.5×10^{-9}
$t_{1/2}$ (h)	2.89 (25.2%)	3.03 (29.7%)	3.33 (29.6%)
GMR	*Control*	1.05 (0.96-1.15)	1.15 (1.00-1.33)
P-value		0.50	0.10
**1'-OH-midazolam (N=11)**			
C_{max} (ng/mL)	2.94 (33.4%)	2.99 (36.6%)	3.69 (31.0%)
GMR	*Control*	1.02 (0.86-1.21)	1.25 (1.04-1.51)
P-value		>0.99	0.043
T_{max} (h)	1.0 (0.5-1.5)	1.0 (0.5-1.0)	1.5 (1.0-1.5)
P-value	*Control*	0.73	0.017
$AUC_{0\text{-}23h}$ (ng×h/mL)	6.59 (30.5%)	6.65 (30.2%)	11.4 (30.3%)
GMR	*Control*	1.01 (0.88-1.15)	1.73 (1.46-2.05)
P-value		>0.99	6.2×10^{-5}
$AUC_{0\text{-}\infty}$ (ng×h/mL)	6.90 (29.4%)	6.94 (29.9%)	11.5 (29.2%)
GMR	*Control*	1.01 (0.87-1.16)	1.67 (1.41-1.98)
P-value		>0.99	9.9×10^{-5}
$t_{1/2}$ (h)	1.80 (31.4%)	1.63 (31.5%)	2.38 (46.3%)
GMR	*Control*	0.90 (0.71-1.15)	1.32 (0.93-1.89)
P-value		0.75	0.22

**Simvastatin (N=11)[11]**			
C_{max} (ng/mL)	2.11 (93.2%)	2.08 (72.7%)	17.3 (70.5%)
GMR	*Control*	0.98 (0.66-1.46)	8.18 (6.50-10.3)
P-value		>0.99	3.7×10^{-9}
T_{max} (h)	2.0 (1.0-4.0)	1.5 (1.5-8.0)	2.0 (1.5-4.0)
P-value	*Control*	0.33	>0.99
AUC_{0-23h} (ng×h/mL)	14.1 (62.1%)	12.2 (63.0%)	70.4 (65.5%)
GMR	*Control*	0.87 (0.67-1.12)	5.00 (4.25-5.88)
P-value		0.49	1.6×10^{-9}
$AUC_{0-\infty}$ (ng×h/mL) (N=10)[12]	16.7 (66.0%)	14.4 (82.7%)	73.9 (68.9%)
GMR	*Control*	0.86 (0.60-1.25)	4.42 (3.72-5.26)
P-value		0.78	2.4×10^{-8}
$t_{1/2}$ (h) (N=10)[12]	8.51 (20.0%)	6.15 (104%)	4.78 (19.8%)
GMR	*Control*	0.72 (0.38-1.38)	0.56 (0.45-0.69)
P-value		0.57	3.4×10^{-4}
**Simvastatin acid (N=11)[11]**			
C_{max} (ng/mL)	0.73 (93.1%)	0.65 (77.3%)	3.04 (118%)
GMR	*Control*	0.89 (0.69-1.15)	4.18 (3.11-5.61)
P-value		0.65	1.6×10^{-6}
T_{max} (h)	4.0 (1.5-6.0)	4.0 (1.5-6.0)	4.0 (4.0-8.0)
P-value	*Control*	>0.99	0.67
AUC_{0-23h} (ng×h/mL)	5.52 (123%)	5.16 (127%)	22.6 (108%)
GMR	*Control*	0.94 (0.72-1.22)	4.10 (2.95-5.70)
P-value		>0.99	4.8×10^{-6}

**Simvastatin acid (N=11)[11]**			
$AUC_{0-\infty}$ (ng×h/mL) (N=7)[13]	11.0 (65.1%)	9.92 (126%)	36.2 (75.2%)
GMR	*Control*	0.90 (0.54-1.52)	3.29 (2.05-5.27)
P-value		>0.99	0.0016
$t_{1/2}$ (h) (N=7)[13]	5.95 (49.8%)	6.08 (61.5%)	4.07 (32.5%)
GMR	*Control*	1.02 (0.58-1.79)	0.68 (0.44-1.07)
P-value		>0.99	0.17

[1] Calculation of $t_{1/2}$ for paraxanthine was not possible for three study participants because there was insufficient number of data points in the elimination phase in at least one study phase.
[2] CYP2B6 phenotypes were the following: 7 normal, 3 intermediate and 1 poor metabolizer, who was excluded from the statistical analyses regarding bupropion and hydroxybupropion.
[3] Calculation of $t_{1/2}$ for hydroxybupropion was not possible for one study participant because there was insufficient number of data points in the elimination phase in at least one study phase.
[4] CYP2C9 phenotypes were the following: 9 normal and 2 intermediate metabolizers.
[5] CYP2C19 phenotypes were the following: 1 ultrarapid, 2 rapid, 2 normal, 5 intermediate and 1 poor metabolizer, who was excluded from the statistical analyses regarding omeprazole and its metabolites.
[6] Calculation of $t_{1/2}$ for omeprazole was not possible for three study participants because there was insufficient number of data points in the elimination phase in at least one study phase.
[7] Calculation of $t_{1/2}$ for 5'-hydroxyomeprazole was not possible for two study participants because there was insufficient number of data points in the elimination phase in at least one study phase.
[8] Calculation of $t_{1/2}$ for omeprazole sulfone was not possible for three study participants because there was insufficient number of data points in the elimination phase in at least one study phase.
[9] CYP2D6 phenotypes were the following: 9 normal and 2 intermediate metabolizers.
[10] Calculation of $t_{1/2}$ for dextromethorphan was not possible for one study participant because there was insufficient number of data points in the elimination phase in at least one study phase.
[11] OATP1B1 phenotypes were the following: 7 normal, 1 decreased and 3 poor function phenotypes. All phenotypes were included in statistical analyses.
[12] Calculation of $t_{1/2}$ for simvastatin was not possible for one study participant because there was insufficient number of data points in the elimination phase in at least one study phase.

[13] Calculation of $t_{1/2}$ for simvastatin acid was not possible for four study participants because there was insufficient number of data points in the elimination phase in at least one study phase.

Table S3. The genotypes, activity scores, and predicted phenotypes of CYP enzymes, *SLCO1B1* (OATP1B1), and *ABCG2* (BCRP) in the 11 study participants. For some genotypes, two alternative genotypes could not be distinguished and both alternatives are shown.

Enzyme/transporter	Genotype	Activity score*	Phenotype
CYP2B6	*6/*6 (N=1)		PM
	*1/*6 or *4/*9 (N=3)		IM
	*1/*1 (N=6)		NM
	*1/*5 (N=1)		
CYP2C9	*1/*3 (N=2)	1.0	IM
	*1/*1 (N=9)	2.0	NM
CYP2C19	*2/*2 (N=1)		PM
	*1/*2 (N=3)		IM
	*2/*17 (N=2)		
	*1/*1 (N=2)		NM
	*1/*17 (N=2)		RM
	*17/*17 (N=1)		UM
CYP2D6	*1/*6 (N=1)	1.0	IM
	*2/*5 (N=1)	1.0	
	*1/*1 (N=3)	2.0	NM
	*1/*2 (N=6)	2.0	
CYP3A4	*1/*1 (N=11)		NM
CYP3A5	*3/*3 (N=9)		PM
	*1/*3 (N=2)		IM
OATP1B1	*15/*15 (N=3)		PF

	*1/*5 (N=1)	DF
	*1/*1 (N=3)	NF
	*1/*37 (N=2)	
	*1/*14 or *4/*37 (N=2)	
BCRP	c.421AA (N=1)	PF
	c.421CA (N=2)	DF
	c.421CC (N=8)	NF

DF, decreased function, IM, intermediate metabolizer, NF, normal function, NM, normal metabolizer, PF, poor function, PM, poor metabolizer, RM, rapid metabolizer, UM, ultrarapid metabolizer.

*References:

Theken, K.N., et al., Clinical Pharmacogenetics Implementation Consortium Guideline (CPIC) for CYP2C9 and Nonsteroidal Anti-Inflammatory Drugs. *Clin Pharmacol Ther.* **108(2)**, 191-200 (2020).

Bell, C.G., et al. Clinical Pharmacogenetics Implementation Consortium (CPIC) guideline for CYP2D6 genotype and use of ondansetron and tropisetron. *Clin Pharmacol Ther.* **102(2)**, 213-218 (2017).

Table S4. The 2-hour and 4-hour metabolic ratios and AUC ratios of CYP indices (CYP1A2: paraxanthine/caffeine, CYP2B6: OH-bupropion/bupropion, CYP2C9: 4'-OH-flurbiprofen/flurbiprofen, CYP2C19: 5'-OH-omeprazole/omeprazole, CYP2D6: dextrorphan/dextromethorphan, CYP3A4: 1'-OH-midazolam/midazolam and omeprazole sulfone/omeprazole) in healthy subjects as geometric mean values (with geometric CV) in the three study phases. Geometric mean ratios (GMR) with 90% confidence intervals compared to the control phase are indicated on the rows below each pharmacokinetic variable.

	Water (control)	Lingonberry powder	Grapefruit juice
**CYP1A2 (N=11)**			
2-hour metabolic ratio	0.39 (56.3%)	0.45 (37.0%)	0.31 (43.0%)
GMR	*Control*	1.14 (0.82-1.59)	0.79 (0.61-1.03)
P-value		0.81	0.15
4-hour metabolic ratio	0.56 (40.5%)	0.65 (29.0%)	0.43 (36.0%)
GMR	*Control*	1.15 (0.90-1.45)	0.75 (0.62-0.92)
P-value		0.46	0.020
AUC_{0-4h} ratio	0.40 (54.7%)	0.48 (41.9%)	0.35 (51.7%)
GMR	*Control*	1.18 (0.85-1.64)	0.88 (0.66-1.18)
P-value		0.57	0.70
AUC_{0-12h} ratio	0.61 (36.7%)	0.67 (29.3%)	0.49 (35.5%)
GMR	*Control*	1.09 (0.89-1.34)	0.80 (0.66-0.98)
P-value		0.72	0.068
$AUC_{0-\infty}$ ratio (N=8)[1]	1.04 (21.0%)	1.02 (25.9%)	0.98 (28.5%)
GMR	*Control*	0.98 (0.85-1.14)	0.94 (0.80-1.10)
P-value		>0.99	0.73
**CYP2B6[2] (N=10)**			
2-hour metabolic ratio	1.61 (64.9%)	1.62 (46.7%)	0.77 (65.5%)
GMR	*Control*	1.00 (0.74-1.37)	0.47 (0.33-0.67)
P-value		>0.99	0.0018

**CYP2B6[2] (N=10)**			
4-hour metabolic ratio	3.90 (61.3%)	3.89 (45.5%)	2.01 (62.0%)
GMR	*Control*	1.00 (0.76-1.31)	0.51 (0.34-0.77)
P-value		>0.99	0.0091
$AUC_{0\text{-}4h}$ ratio	1.88 (60.0%)	1.90 (49.3%)	0.94 (66.5%)
GMR	*Control*	1.01 (0.81-1.25)	0.50 (0.37-0.67)
P-value		>0.99	9.1×10^{-4}
$AUC_{0\text{-}23h}$ ratio	5.77 (48.5%)	5.73 (42.2%)	3.31 (54.7%)
GMR	*Control*	0.99 (0.85-1.16)	0.57 (0.45-0.74)
P-value		>0.99	0.0016
$AUC_{0\text{-}\infty}$ ratio (N=9)[3]	12.4 (40.6%)	11.8 (49.0%)	10.2 (62.4%)
GMR	*Control*	0.95 (0.84-1.07)	0.82 (0.59-1.13)
P-value		0.72	0.37
**CYP2C9[4] (N=11)**			
2-hour metabolic ratio	0.060 (34.9%)	0.055 (27.6%)	0.038 (35.4%)
GMR	*Control*	0.91 (0.76-1.09)	0.64 (0.52-0.78)
P-value		0.56	0.0011
4-hour metabolic ratio	0.064 (33.4%)	0.057 (26.2%)	0.053 (29.1%)
GMR	*Control*	0.89 (0.75-1.06)	0.82 (0.71-0.95)
P-value		0.35	0.023
$AUC_{0\text{-}4h}$ ratio	0.056 (31.0%)	0.052 (24.7%)	0.039 (30.2%)
GMR	*Control*	0.93 (0.81-1.06)	0.69 (0.59-0.81)
P-value		0.44	6.6×10^{-4}
$AUC_{0\text{-}23h}$ ratio	0.065 (28.4%)	0.062 (23.5%)	0.050 (29.1%)
GMR	*Control*	0.96 (0.86-1.07)	0.78 (0.69-0.87)
GMR		0.92	0.0013

**CYP2C9[4] (N=11)**			
$AUC_{0-\infty}$ ratio	0.068 (24.4%)	0.067 (19.7%)	0.054 (28.8%)
GMR	*Control*	0.98 (0.88-1.09)	0.80 (0.72-0.89)
GMR		>0.99	0.0021
**CYP2C19[5] (N=10)**			
2-hour metabolic ratio (N=9)[6]	1.36 (60.1%)	1.53 (63.2%)	0.48 (61.3%)
GMR	*Control*	1.13 (0.90-1.41)	0.35 (0.26-0.48)
P-value		0.50	1.1×10^{-4}
4-hour metabolic ratio	2.49 (81.8%)	2.55 (95.3%)	0.72 (63.1%)
GMR	*Control*	1.02 (0.73-1.44)	0.29 (0.22-0.37)
P-value		>0.99	2.8×10^{-6}
AUC_{0-4h} ratio	1.28 (43.4%)	1.25 (49.1%)	0.51 (47.8%)
GMR	*Control*	0.98 (0.88-1.08)	0.40 (0.32-0.50)
P-value		>0.99	9.7×10^{-6}
AUC_{0-23h} ratio	1.45 (43.0%)	1.41 (47.3%)	0.63 (46.2%)
GMR	*Control*	0.97 (0.89-1.06)	0.43 (0.36-0.52)
P-value		>0.99	8.4×10^{-6}
$AUC_{0-\infty}$ ratio (N=7)[7]	1.45 (43.6%)	1.42 (49.1%)	0.64 (54.1%)
GMR	*Control*	0.98 (0.88-1.10)	0.44 (0.39-0.50)
P-value		>0.99	7.5×10^{-6}
**CYP2D6[8] (N=11)**			
2-hour metabolic ratio	3.44 (69.0%)	3.37 (65.6%)	2.84 (72.6%)
GMR	*Control*	0.98 (0.79-1.21)	0.83 (0.57-1.19)
P-value		>0.99	0.55
4-hour metabolic ratio	2.63 (67.6%)	2.47 (70.7%)	2.18 (79.2%)
GMR	*Control*	0.94 (0.75-1.17)	0.83 (0.54-1.29)
P-value		>0.99	0.73

**CYP2D6[8] (N=11)**			
$AUC_{0\text{-}4h}$ ratio	3.32 (67.2%)	3.36 (73.3%)	2.78 (76.8%)
GMR	*Control*	1.01 (0.83-1.24)	0.84 (0.58-1.22)
P-value		>0.99	0.64
$AUC_{0\text{-}23h}$ ratio	2.57 (95.5%)	2.39 (116%)	1.92 (98.0%)
GMR	*Control*	0.93 (0.71-1.21)	0.75 (0.46-1.23)
P-value		>0.99	0.44
$AUC_{0\text{-}\infty}$ ratio (N=10)[9]	2.03 (69.7%)	1.82 (85.4%)	1.78 (98.9%)
GMR	*Control*	0.90 (0.67-1.20)	0.88 (0.57-1.34)
P-value		0.85	>0.99
**CYP3A4 (Midazolam, N=11)**			
2-hour metabolic ratio	0.54 (28.2%)	0.57 (33.9%)	0.38 (37.2%)
GMR	*Control*	1.05 (0.92-1.20)	0.71 (0.62-0.82)
P-value		0.79	5.9×10^{-4}
4-hour metabolic ratio	0.53 (28.1%)	0.51 (32.3%)	0.38 (39.8%)
GMR	*Control*	0.97 (0.86-1.10)	0.72 (0.61-0.85)
P-value		>0.99	0.0031
$AUC_{0\text{-}4h}$ ratio	0.51 (25.0%)	0.50 (27.4%)	0.35 (35.4%)
GMR	*Control*	0.99 (0.88-1.11)	0.68 (0.60-0.78)
P-value		>0.99	1.7×10^{-4}
$AUC_{0\text{-}23h}$ ratio	0.45 (26.5%)	0.43 (27.8%)	0.32 (35.7%)
GMR	*Control*	0.97 (0.85-1.11)	0.72 (0.63-0.84)
P-value		>0.99	0.0012
$AUC_{0\text{-}\infty}$ ratio	0.47 (27.8%)	0.45 (27.9%)	0.33 (37.1%)
GMR	*Control*	0.97 (0.84-1.12)	0.69 (0.59-0.81)
P-value		>0.99	6.2×10^{-4}

**CYP3A4[5] (Omeprazole, N=10)**			
2-hour metabolic ratio (N=8)[10]	0.50 (72.0%)	0.59 (55.6%)	0.13 (65.1%)
GMR	*Control*	1.17 (0.81-1.71)	0.26 (0.20-0.35)
P-value		0.69	2.2×10^{-5}
4-hour metabolic ratio (N=9)[11]	1.44 (85.3%)	1.62 (140%)	0.40 (80.7%)
GMR	*Control*	1.12 (0.61-2.07)	0.28 (0.20-0.38)
P-value		>0.99	3.4×10^{-5}
AUC_{0-4h} ratio (N=9)[11]	0.54 (25.5%)	0.52 (27.9%)	0.18 (48.8%)
GMR	*Control*	0.96 (0.75-1.24)	0.33 (0.25-0.42)
P-value		>0.99	1.8×10^{-5}
AUC_{0-23h} ratio	0.79 (21.9%)	0.74 (22.6%)	0.47 (54.4%)
GMR	*Control*	0.94 (0.81-1.09)	0.60 (0.47-0.76)
P-value		0.77	0.0022
$AUC_{0-\infty}$ ratio (N=7)[7]	0.76 (23.7%)	0.80 (20.1%)	0.45 (70.8%)
GMR	*Control*	1.05 (0.93-1.18)	0.59 (0.39-0.88)
P-value		0.73	0.038

[1] Calculation of $t_{1/2}$ for paraxanthine was not possible for three study participants because there was insufficient number of data points in the elimination phase in at least one study phase.
[2] CYP2B6 phenotypes were the following: 7 normal, 3 intermediate and 1 poor metabolizer, who was excluded from the statistical analyses regarding bupropion and hydroxybupropion.
[3] Calculation of $t_{1/2}$ for hydroxybupropion was not possible for one study participant because there was insufficient number of data points in the elimination phase in at least one study phase.
[4] CYP2C9 phenotypes were the following: 9 normal and 2 intermediate metabolizers.
[5] CYP2C19 phenotypes were the following: 1 ultrarapid, 2 rapid, 2 normal, 5 intermediate and 1 poor metabolizer, who was excluded from the statistical analyses regarding omeprazole and its metabolites.
[6] For one participant 2-hour metabolic ratio could not be calculated as 5'-hydroxyomeprazole and omeprazole concentrations were below limit of quantification at the 2-hour sampling point.
[7] Calculation of $t_{1/2}$ for omeprazole was not possible for three study participants because there was insufficient number of data points in the elimination phase in at least one study phase.

[8] CYP2D6 phenotypes were the following: 9 normal and 2 intermediate metabolizers.
[9] Calculation of $t_{1/2}$ for dextromethorphan was not possible for one study participants because there was insufficient number of data points in the elimination phase in at least one study phase.
[10] For two participants 2-hour metabolic ratio could not be calculated as omeprazole sulfone concentrations were below limit of quantification at the 2-hour sampling point.
[11] For one participant 4-hour metabolic ratio and $AUC_{0\text{-}4h}$ could not be calculated as omeprazole sulfone concentrations were below limit of quantification until the 8-hour sampling point.

Table S5. Metabolic ratios at 8.00 a.m. (0-hour metabolic ratio) and AUC_{0-4h} ratios (based on concentrations from pretreatment administration at 8.00 a.m. to sampling time point at 12 a.m. on study days) of solanidine and its metabolites M430 and M444 in healthy subjects in the grapefruit juice pretreatment phase compared to the control phase. Variables are expressed as geometric mean values (with geometric CV). Geometric mean ratios (GMR) (with 90% confidence intervals) compared to the control phase are indicated on the rows below each variable. Four participants, who did not have detectable solanidine concentrations in at least one of the phases, were excluded from the analysis.

	**Water (control)**	**Grapefruit juice**
**M430/solanidine (N=7)**		
0-hour metabolic ratio	20.4 (85.5%)	30.6 (96.0%)
GMR	*Control*	1.49 (0.87-2.58)
P-value		0.24
AUC_{0-4h} ratio	18.8 (87.9%)	28.5 (115%)
GMR	*Control*	1.52 (0.94-2.47)
P-value		0.16
**M444/solanidine (N=7)**		
0-hour metabolic ratio	42.5 (73.5%)	60.5 (119%)
GMR	*Control*	1.42 (0.88-2.31)
P-value		0.24
AUC_{0-4h} ratio	37.2 (70.0%)	56.5 (117%)
GMR	*Control*	1.52 (1.02-2.25)
P-value		0.083

Table S6. Metabolic ratios at 8.00 a.m. (0-hour metabolic ratio) and AUC_{0-4h} ratios (based on concentrations from pretreatment administration at 8.00 a.m. to sampling time point at 12 a.m. on study days) of solanidine and its metabolites M430 and M444 in healthy subjects in the lingonberry powder pretreatment phase compared to the control phase. Variables are expressed as geometric mean values (with geometric CV). Geometric mean ratios (GMR) (with 90% confidence intervals) compared to the control phase are indicated on the rows below each variable. Four participants, who did not have detectable solanidine concentrations in at least one of the phases, were excluded from the analysis.

	Water (control)	Lingonberry powder
**M430/solanidine (N=7)**		
0-hour metabolic ratio	20.2 (83.3%)	15.2 (66.8%)
GMR	*Control*	0.75 (0.29-1.92)
P-value		0.97
AUC_{0-4h} ratio	18.5 (84.9%)	14.5 (74.7%)
GMR	*Control*	0.78 (0.32-1.94)
P-value		>0.99
**M444/solanidine (N=7)**		
0-hour metabolic ratio	38.0 (58.9%)	30.2 (74.0%)
GMR	*Control*	0.79 (0.35-1.82)
P-value		>0.99
AUC_{0-4h} ratio	33.2 (52.0%)	27.1 (87.2%)
GMR	*Control*	0.82 (0.36-1.87)
P-value		>0.99
